# Financial impact of participating in oncology clinical trials in a tertiary hospital in Spain

**DOI:** 10.1186/s12913-026-15148-z

**Published:** 2026-07-18

**Authors:** Sergio Mosquera-Ferrer, Emilio Vargas-Castrillón, Macarena Torrego-Ellacuría

**Affiliations:** 1 Clinical Pharmacology, Hospital Clínico San Carlos, Calle del Prof Martín Lagos, S/N, Madrid, 28040 Spain; 2 Clinical Pharmacology; Pharmacology and Toxicology, Hospital Clínico San Carlos, Universidad Complutense de Madrid, Instituto de Investigación Sanitaria San Carlos (IdISSC), Madrid, Spain; 3 Innovation Unit, Hospital Clinico San Carlos, IdISSC, Madrid, Spain

**Keywords:** Cancer, Clinical trials, Investigational drug, Cost avoidance, Cost savings

## Abstract

**Background:**

This study aimed to evaluate the financial impact, including medication-related costs and other costs, of conducting randomized controlled trials (RCTs) in the oncology department of a tertiary hospital.

**Methods:**

This retrospective study included all clinical trials conducted in the oncology department from January 2018 to December 2023, regardless of the phase of clinical research or type of sponsor. The total avoided cost was calculated as the drug cost avoided minus other direct medical cost for activities performed in the hospital using data from the official publication of the autonomous community health authority.

**Results:**

During the observation period, 197 clinical trials actively recruited 1,198 patients, 757 of whom completed their participation within the established timeframe and were dispensed trial medications. Drug cost avoidance totaled €25,899,148, with a median (interquartile range [IQR]) by trial of €61,034 (€5,280, €235,588). Both industry-sponsored and noncommercially sponsored studies were associated with drug cost avoidance with an absolute (relative) contribution to the total cost avoidance of €23,134,649 (89.3%) and €2,764,499 (10.7%), respectively. Other direct medical cost for activities performed in the hospital €3,274,099.80. The total cost avoidance was €22,625,048. There were large variations in cost avoidance depending on the type of tumor.

**Conclusions:**

Although there are large variations across types of tumors, overall, clinical trials performed at the medical oncology department of a tertiary university hospital are associated with high cost avoidance and could contribute to reducing hospital expenditures for the management of cancer.

**Trial registration:**

Not applicable.

**Supplementary Information:**

The online version contains supplementary material available at https://doi.org/10.1186/s12913-026-15148-z.

## Background

The International Agency for Research on Cancer estimated that, in 2022, there were 20 million new cases of cancer and 9.7 million deaths from cancer worldwide, and these figures are expected to increase to 35 million new cases by 2050 [1]. In addition to the individual burden, cancer is associated with high economic costs, which have been estimated globally in over 25 trillion international dollars globally from 2020 to 2050 [2]. In 2016, cancer was estimated to be responsible for 4.6% of total health care spending in the United States ($123.8 billion from total spending of $2705.6 billion globally) [3].

However, life expectancy is increasing among people with cancer [4], and after public health interventions, pharmaceuticals are estimated to be responsible for 35% of that increase [5]. Randomized controlled trials (RCTs), the gold standard for evaluating the effect and value of an intervention, are responsible for these achievements.

In addition to the benefits to individuals, RCTs may contribute to reducing health care expenditures. The revised guidelines for good clinical practice and local regulations in Europe establish that the sponsor of a trial is responsible for supplying the investigational products [6]. Therefore, RCTs are associated with cost savings. Several studies have shown that participation in RCTs on oncology is associated with cost savings due to drug cost avoidance [7–11]. These savings, although larger in pharmaceutical industry-sponsored studies, also occurred in academic-sponsored trials [8, 11]. Studies evaluating the impact of RCTs in oncology and reporting cost savings in Spain have also been reported [12–18]. However, the extent of the financial impact of RCTs depends on the type of tumor [11, 14]^,^ and some Spanish studies have been conducted on specific tumors [12, 13]. In addition, performing a clinical trial is associated with costs other than those related to medication [10]; to the best of our knowledge, only one study conducted in Spain has included those costs, and it had a very limited number of trials and patients [16].

This study aimed to evaluate the financial impact of conducting RCTs in the oncology department of a tertiary hospital.

## Methods

This retrospective study was conducted in the medical oncology department of a tertiary hospital in Madrid (Spain). The study was approved by the ethics committee of the Hospital Clínico de San Carlos (Madrid, Spain; code 23/499-E) and was conducted according to the principles of the Declaration of Helsinki. Owing to the safeguarding of the confidentiality of personal data, this study involved minimal risk; furthermore, informed consent was impractical to obtain because it would have required a disproportionate amount of effort and resources relative to the research activities, so the requirement to obtain informed consent was waived.

### Selection criteria

We included all clinical trials conducted in the oncology department from January 2018 to December 2023, regardless of the research phase or the type of sponsor. Observational studies and clinical trials that did not recruit patients within that timeframe were excluded. We included all patients who were included in the trial within that timeframe, provided that they had been dispensed at least one unit of medication and had completed their participation before January 1, 2024.

### Source of data and information recorded

We used several hospital databases to obtain the information required for our study.

The clinical trial database of the medical oncology department provides specific information on the trials that were included in our analysis and on the patients who were included in each trial. From this database, we obtained information on the characteristics of each trial, including its design, phase, oncologic condition, and type of sponsor; whether it was multicenter; and the number of patients included. We also obtained patient identification needed for requesting information from other databases.

The pharmacy department’s medicine database provided specific information on the medicines that were dispensed, including its pharmaceutical form, dosage, route of administration, and dates of dispensation, together with whether the medicine was approved at the time of the trial participation according to its summary of product characteristics for the condition under investigation in that clinical trial.

We obtained information on demographics, diagnostic tests performed in-house or externally, and medical visits from the admissions department. The medical visits included first and follow-up visits to the medical oncologist and/or genetic counseling, telephone visits with the medical oncologist, nursing visits, visits to the chemotherapy day unit, and visits to the hospital ward.

The costs of the medicines, diagnostic tests including imaging tests and visits were obtained from the official publication of the autonomous community health authority [19]. We did not use information from the hospital’s cost unit owing to the lack of information on some costs included in our calculations (e.g. cost of chemotherapy day unit visits).

### Statistical analysis

No sample size estimation was performed; instead, we included all clinical trials conducted by the medical oncology department during the abovementioned time period.

For the trial and patient characteristics, we used the following descriptive statistics: means and standard deviations or medians and interquartile ranges for quantitative variables and absolute and relative frequencies for qualitative variables.

Using the aforementioned sources, the cost of participating in a clinical trial was calculated as the cost of medicines plus other direct medical costs. The cost of medicines was calculated as the selling price of the medicine at the time of the analysis (2023) multiplied by the number of dispensed units; it was only calculated for drugs that were being marketed at the time of study initiation. Other direct medical costs included the cost of complementary and imaging diagnostic tests plus the cost of medical visits, which was calculated by multiplying the number of tests or visits by the corresponding costs at the time of the analysis (2023).

Drug cost avoidance was calculated as the cost of all investigational drugs and/or comparators, provided that the trial was in an advanced phase (i.e., phase 2, phase 3, or later) and was indicated for the condition being investigated at the time of the study. The total avoided cost was calculated as the drug cost avoided minus other direct medical costs for activities performed in the hospital (i.e. activities contracted out were excluded from this calculation). Regarding other direct medical costs, it is important to note that it was not possible to reliably estimate the standard-of-care activity costs for each patient in the absence of trial participation. Therefore, we adopted a conservative approach (i.e. it underestimates total cost avoidance) by attributing the full direct medical cost to trial participation when calculating total cost avoidance.

To integrate the information from the databases and descriptive analyses, we used the Microsoft tool Power BI vs. 2.1.

## Results

### Studies included and their descriptions

**Fig. 1:**
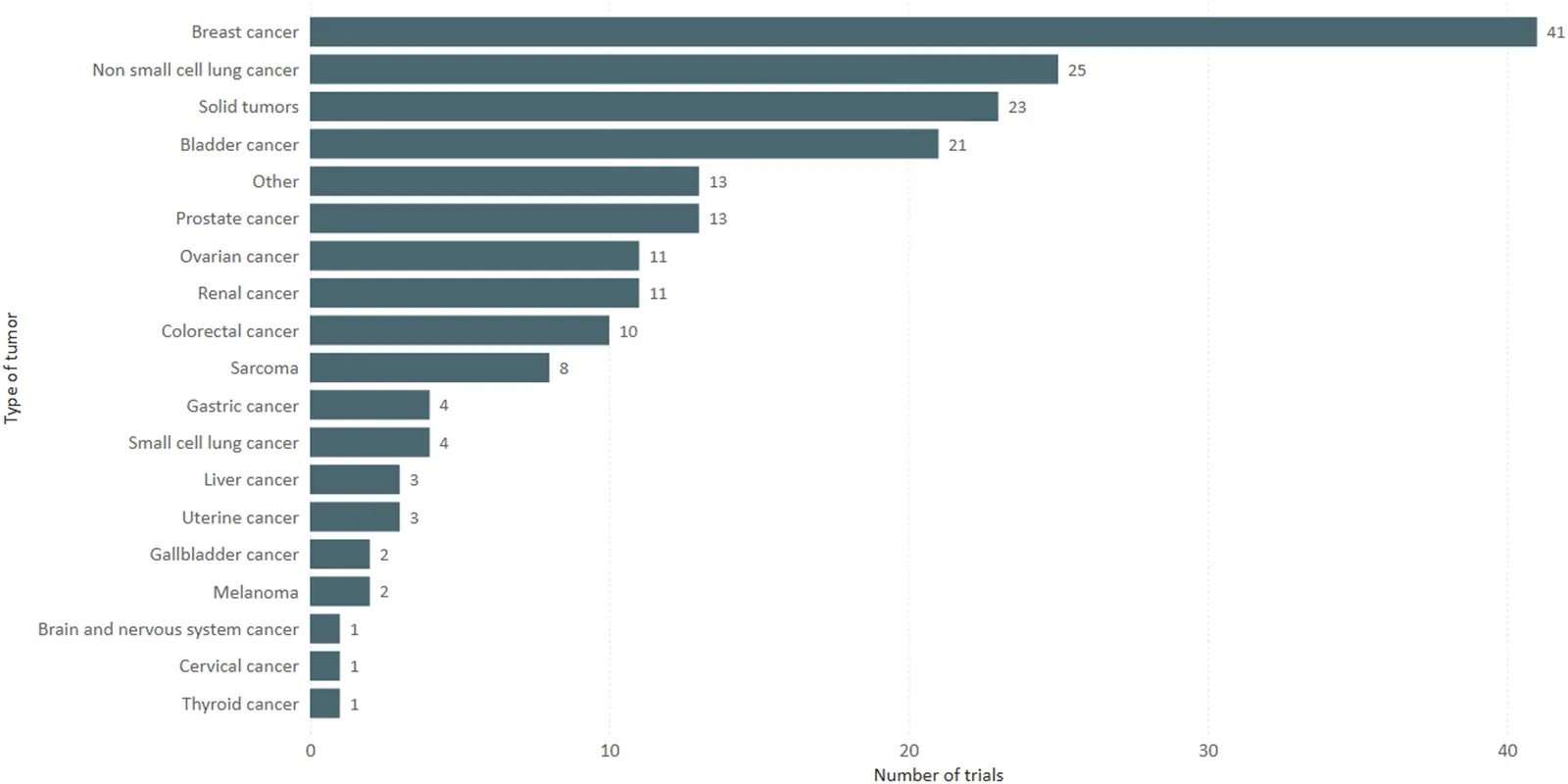
Distribution of number of clinical trials by type of tumor

During the observation period, 197 clinical trials actively recruited patients (Supplementary Figure 1). Trials phase included phase 3 (*n* = 104, 52.8%), phase 2 (*n* = 52, 26.4%), phase 1–2 (*n* = 19, 9.6%), phase 1 (*n* = 15, 7.6%), phase 2–3 (*n* = 6, 3.1%) and phase IV (*n* = 1, 0.5%). The most frequently involved tumors were breast cancer (*n* = 41, 20.8%), non-small cell lung cancer (*n* = 25, 12.7%), multiple solid tumors (*n* = 23, 11.7%), and bladder cancer (*n* = 21, 10.7%) (Fig. 1). The pharmaceutical industry sponsored 153 (77.7%) trials, and 44 (22.3%) trials were sponsored by noncommercial organizations; a detailed distribution of sponsors is presented in Supplementary Figure 2. Most trials were multicenter and international (*n* = 174, 88.3%), and most were randomized trials (*n* = 141, 71.57%).

There were 1,198 patients included in these trials, 757 of whom completed their participation within the established timeframe and were dispensed trial medications (Supplementary Figure 1). Sex was evenly distributed, with 392 (51.8%) women and 365 (48.2%) men, and the mean age was 64.8 (12.2) years.

### Medicines and resource use

Among the 757 patients, 29,486 units of medication were dispensed. The ten most frequently dispensed drugs were durvalumab (2,536 units), nivolumab (1,560 units), bevacizumab (1,517 units), pembrolizumab (1,467 units), olaparib (1,277 units), lenvatinib (1016 units), paclitaxel (964 units), atezolizumab (826 units), cetuximab (779 units) and letrozole (699 units); these drugs accounted for 42.9% of the dispensed units. Of the 29,486 units of medication dispensed, 24,858 (84.3%) corresponded to 84 medicines approved for the condition being investigated at the time of the study. Among the latter units, 13,404 (53.9%) were assigned to the experimental arm, 7,661 (30.8%) to the experimental and control treatment arms, and 3,793 (15.3%) to the control arm.

Overall, 16,583 complementary tests were performed; 13,804 (83.2%) were performed in-house, and 2,779 (16.8%) were performed externally. The most frequently performed tests were laboratory tests (hematologic and biochemical) (*n* = 11,427, 68.9%), computed tomography (*n* = 2,679, 16.2%; 565 in-house and 2,112 externally), gammagraphy (*n* = 565, 3.4%), and radiography (*n* = 427, 2.6%) (Supplementary Table 1).

There were 20,985 medical visits, mostly chemotherapy day units (*n* = 13,499, 64.3%), nursing visits (*n* = 5195, 24.8%), and medical oncologist follow-up visits (*n* = 1284, 6.1%) (Supplementary Table 2).

### Cost analysis

The total cost of medicines was €43,388,876, which was higher for ovarian cancer (€9,729,281), non-small cell lung cancer (€7,116,724), renal cancer (€5,438,490), and breast cancer (€5,217,459) (Supplementary Figure 3). The cost was greater for phase III trials (31,691,107) and phase II trials (7,250,225). The cost was greater for interventional groups (€33,874,645).

Other direct medical costs accounted for €3,617,584 according to the local health authorities’ official prices, which were higher for phase III studies (€2,308,339) and breast cancer (€588,374). The largest contributor to other direct medical costs was visits to the chemotherapy day unit (€2,024,550). When accounting only for internal activities, other direct medical costs totaled €3,274,099.80.

The total cost was €47,006,460 overall and a median (IQR) by trial of €80,004 (€15,777, €260,606) and was higher for phase III trials (€33,999,447 overall, 71,128.55 per patient) than for phase II trials (€7,959,936.29 overall, 48,242.04 per patient). Overall, costs were higher for ovarian cancer (€10,078,831), non-small cell lung cancer (€7,499,220), breast cancer (€5,805,833), and renal cancer (€5,642,563) (Table 1). However, in terms of cost per patient, ovarian cancer (€176,822), renal cancer (€170,987), liver cancer (€127,061), and melanoma had higher costs (€104,260) (Table 1).

**Table 1:** Total cost (euros) by type of tumor, overall and per patient

Type of tumor	No. Trials	No. Patients	Overall	Per patient
Ovarian cancer	11	57	10,078,831	176,822
Non-small cell lung cancer	25	107	7,499,220	70,086
Breast cancer	41	157	5,805,833	36,980
Renal cancer	11	33	5,642,563	170,987
Bladder cancer	21	104	4,522,761	43,488
Solid tumors	23	81	3,730,786	46,059
Prostate cancer	13	59	3,120,131	52,884
Other	13	48	1,359,989	28,333
Melanoma	2	11	1,146,864	104,260
Liver cancer	3	8	1,016,486	127,061
Sarcoma	8	21	799,362	38,065
Colorectal cancer	10	25	759,940	30,398
Corpus uteri	3	12	427,636	35,636
Cervical cancer	1	6	395,453	65,909
Small cell lung cancer	4	15	371,064	24,738
Thyroid cancer	1	1	93,146	93,146
Gastric cancer	4	6	42,762	7,127
Gallbladder cancer	2	4	31,877	7,969
Central nervous system	1	2	5,213	2,607

### Cost avoidance

Drug cost avoidance totaled €25,899,148, with a median (IQR) by trial of €61,034 (€5,280–€235,588). Overall, non-small cell lung cancer (€5,698,206), ovarian cancer (€5,283,720), breast cancer (€4,379,542) and renal cancer (€3,289,467) were associated with the greatest drug cost avoidance, whereas per patient cost avoidance was greatest for renal cancer (€99,681), liver cancer (€94,493), ovarian cancer (€92,697) or non-small cell lung cancer (€53,254) (Supplementary Table 3). Phase III trials were associated with the largest drug cost avoidance (€22,709,741 [87.8% of the total]) followed by phase II trials (€1,633,388 [6.3% of the total]); the drug cost avoidance per patient was €47,509.92 for phase III trials and €9,899.32 for phase II trials. Both industry-sponsored and noncommercially sponsored studies were associated with drug cost avoidance (€23,134,649 [89.3%] and €2,764,499 [10.7%], respectively).

**Fig. 2:**
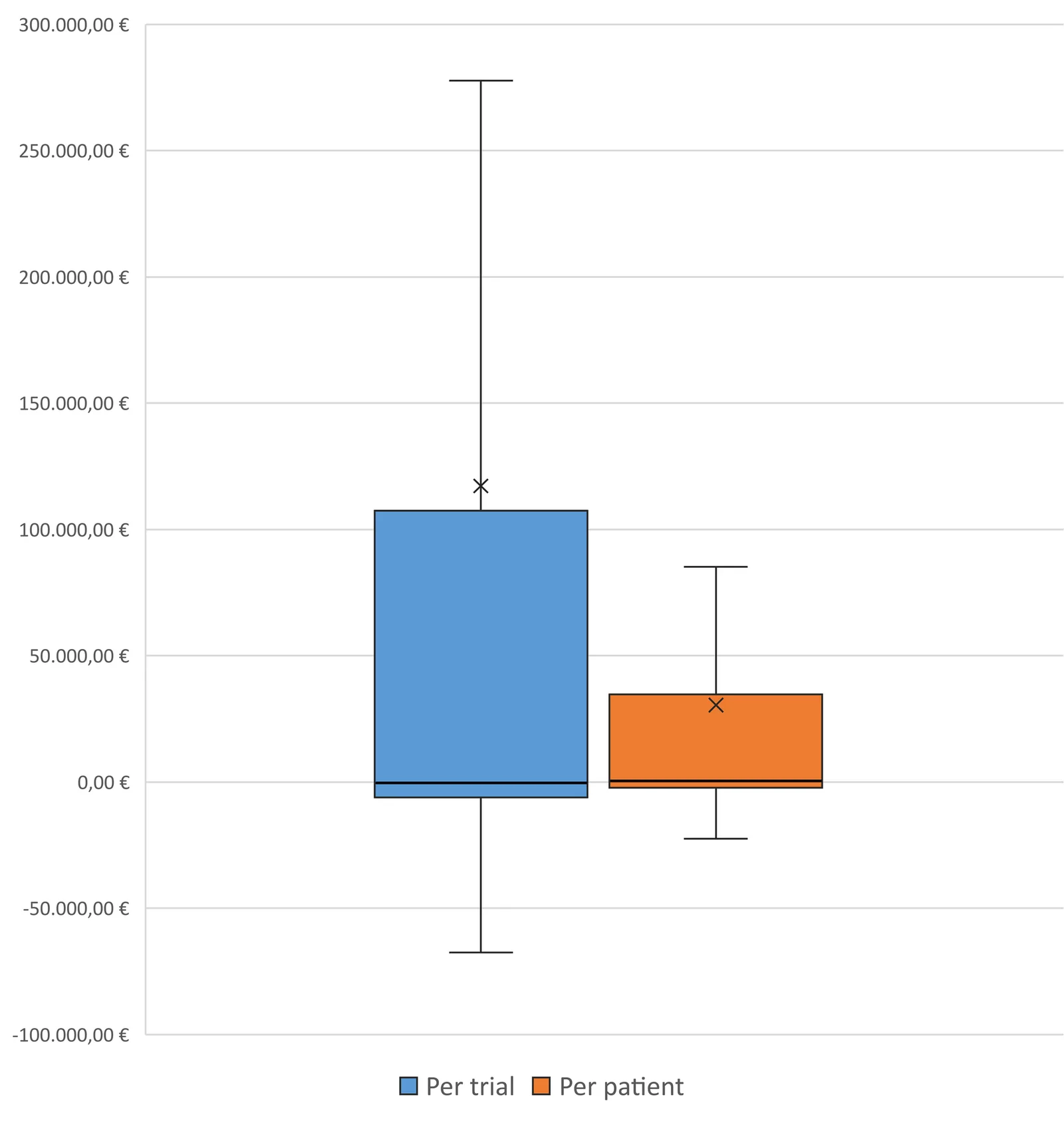
Box plots presenting total cost avoidance per trial and per patient. Positive figures represent cost avoidance, and negative figures represent excess cost. X represents the mean; the horizontal black line represents the median

The total cost avoidance was €22,625,048, varying from an excess cost of €65,785 for trials in patients with sarcoma to cost savings of €5,348,258 for patients with non-small cell lung cancer (Table 2). Per patient, total cost avoidance ranged from an excess cost of €12,146 for thyroid cancer patients to a cost savings of €93,886 for renal cancer patients (Table 2). Phase III trials ranked first in total cost avoidance (€20,529,940), followed by phase II trials (€970,413); the corresponding cost per patient was €42,949.66 and €5,881.29 for phase III and phase II trials, respectively. By trial, the median (IQR) total cost avoidance was €-457 (€-6,170, €104,107) (Fig. 2). By patient, the median (IQR) total cost avoidance was 330 (-2,367, 34,621) (Fig. 2). However, as shown in the figure, the cost avoidance of 50% of the trials above the median was considerably larger than the increased cost (i.e., negative cost avoidance) in the other 50% of the trials below the median.

**Table 2:** Total cost avoidance (euros) by type of tumor, overall and per patient

Type of tumor	No. Trials	No. Patients	Overall	Per patients
Non-small cell lung cancer	25	107	5,348,258	49,984
Ovarian cancer	11	57	4,936,738	86,609
Breast cancer	41	157	3,819,680	24,329
Renal cancer	11	33	3,098,226	93,886
Prostate cancer	13	59	1,617,667	27,418
Bladder cancer	21	104	1,199,199	11,531
Liver cancer	3	8	708,202	88,525
Solid tumors	23	81	531,554	6,562
Melanoma	2	11	457,099	41,554
Other	13	48	378,030	7,876
Colorectal cancer	10	25	302,781	12,111
Cuerpo Uterino	3	12	278,524	23,210
Small cell lung cancer	4	15	61,054	4,070
Gastric cancer	4	6	5,522	920
Central nervous system cancer	1	2	-4,506	-2,253
Thyroid cancer	1	1	-12,146	-12,146
Cervical cancer	1	6	-15,331	-2,555
Gallbladder cancer	2	4	-19,718	-4,929
Sarcoma	8	21	-65,785	-3,133

## Discussion

Our results show that clinical trials performed at the medical oncology department of a tertiary university hospital were associated with high costs but also with large cost savings that were driven by drug cost avoidance. These cost savings were larger for phase III studies and industry-sponsored studies but also occurred in phase II studies and trials sponsored by noncommercial organizations. By tumor type, there was a large variation in the financial impact of trials, with some tumors being associated with a modest excess of cost (e.g., thyroid cancer and gallbladder cancer), while most trials showed large or very large cost savings.

The types of cancer with the greatest number of clinical trials were breast cancer, non-small cell lung cancer, and bladder cancer, and the most common drugs were targeted drugs, which is consistent with the current trends in the clinical development of anticancer drugs [20]. These trials were associated with a large cost (approximately €47 million), for which the major contributor was the cost of medicines (approximately €43 million), with other direct medical costs being a minor contributor (approximately €4 million). There was large variation in the cost of trials depending on the type of tumor, and some tumors contributed to the total cost because of the number of trials and/or patients (i.e., breast cancer) and other trials probably because of the cost of drugs (e.g., ovarian cancer and renal cancer). This explains why the most common tumors in terms of the number of trials were breast cancer, non-small cell lung cancer, and bladder cancer, but among the four with the greatest cost per patient were ovarian cancer and renal cancer.

Despite the large variation in total cost avoidance in relation to the type of tumor, overall, clinical trials in oncology were associated with a total cost avoidance of almost €23 million over a six-year period. This net financial benefit was driven by a large drug cost avoidance of almost €26 million, where phase III trials and industry-sponsored studies contributing the most; however, it is important to note that phase II and noncommercial trials were also associated with drug cost avoidance. Gasperoni et al. [11]. analyzed the results of 244 oncology trials that were conducted by a specialized center and reported similar results, with phase III studies and industry-sponsored studies contributing the most to drug cost avoidance and variations across the tumor types; however, specific figures are not comparable due to the differences in country, methodology, and study period (2018–2020) [11]. In Spain, using a different methodology for calculating drug cost avoided (i.e., it was estimated as the cost that the hospital would have had to bear in the absence of sponsorship), Carreras et al. [18] in Barcelona, Gomez Mediavilla et al. [17] in Santander, and Sánchez Martínez et al. [14] in Murcia reported large cost savings for three other tertiary hospitals, which also varied largely depending on the type of tumor. In fact, in a multivariate analysis, Sanchez Martinez et al. [14] reported that, in addition to the trial phase and intention of treatment (palliative trials, neoadjuvant, and adjuvant), cancer type was a predictor of drug cost avoidance. However, these studies did not consider the cost of the activity. Capdevilla et al. [16], in a study conducted in Pamplona (Spain), included those costs and estimated that medical procedures of clinical trials exceeded the standard of care by 32%, which led to a 25% increase in treatment costs, but the direct funds provided by the sponsor were enough to trade off this excess of cost; consequently, their results are not comparable with ours. In fact, despite the excess of medical procedures, of the 20 trials analyzed by Capdevilla et al. [16], only one was associated with an excess cost. It is important to mention that cost avoidance should not be interpreted solely as a financial benefit, but as a secondary consequence of clinical trial participation, which primarily enables early access to innovative, non-commercialized therapies. Its relevance also depends on its magnitude relative to the department’s drug budget. Additionally, clinical trial activities may generate strategic value, such as facilitating future collaborations with sponsors and strengthening institutional research capacity. Overall, cost avoidance should be considered as one component of the broader value provided by clinical trials.

Our study focused on oncology trials, which have been reported to contribute the most to the cost. In a study conducted in France at a university hospital that included trials from all therapeutic areas, oncology trials contributed to 79% of the cost savings, followed by hepatogastroenterology trials, which contributed to 15% of the cost savings [21]. This study did not include costs other than those of the medications. Therefore, it is not possible to generalize that participation in clinical trials is associated with cost savings regardless of the therapeutic area, and it is important to conduct similar studies in other therapeutic areas where when accounting for activity cost, such studies could be associated with an excess cost. We did not include hematologic oncology trials, which have also been reported to produce large drug cost savings [8, 11]. Importantly, we did not include the potential savings associated with biomarker testing (“pathology cost avoidance”). In a study conducted in Canada that included four trials and 1479 patients, the cost avoided because of pathology testing was over Canadian $4 million (approximately 13% of the total cost avoided) [10]. We did not account for the cost of managing patients in routine clinical practice or potential access to the investigational treatment after the trial funded by the sponsor, which could lead to an underestimation of total cost avoidance. Finally, we did not account for the direct funds provided by the commercial sponsor, which could trade off the activity costs. Unfortunately, detailed information on trial budgets and sponsor expenditures is rarely publicly available because of confidentiality agreements, which limits the ability to perform comprehensive economic evaluations from the sponsor perspective. Overall, these issues could lead to an underestimation of the cost savings of participating in clinical trials. In contrast, the fact that we used the selling price could lead to an overestimation because most drugs have commercial agreements that imply a lower price for hospitals; unfortunately, those agreements are not in the public domain and, therefore, could not be accounted for in our estimations. To bound the effect of using list (selling) prices, we performed a simple sensitivity analysis: applying plausible confidential discounts of 20–40% to drug list prices, the total cost avoidance would range from approximately €17.4 million to €12.3 million (compared with the base-case estimate of €22.6 million), indicating that the net financial benefit, although smaller, would remain substantial across this range. Our estimates also rely on an asymmetric counterfactual: drug costs were fully attributed to the trial, assuming that in its absence patients would have received comparable hospital-funded therapy, whereas other direct medical costs were conservatively attributed in full to trial participation even though part of this care would have been incurred under standard clinical practice; this asymmetry tends to underestimate total cost avoidance. Rankings by tumour type, and particularly per-patient estimates, should be interpreted with caution because some tumour types were represented by a small number of trials and patients, which makes these estimates less stable. Finally, we reported medians with interquartile ranges rather than resampling-based (bootstrap) confidence intervals; given the highly skewed cost distributions and the fact that we analysed the full population of trials conducted at the centre rather than a sample, interval estimation has limited inferential value, although future multicentre studies could incorporate such intervals.

In Spain, the cost of anticancer medicines accounted for less than 30% of the direct cost of cancer in 2008 and over 50% in 2018 [22]. Sanchez Martinez et al. [14], in their study in a tertiary hospital in Murcia (Spain), estimated that drug cost avoidance represented 30% of the annual pharmaceutical expenditure in their center and 0.05% of the annual pharmaceutical expenditure of the Spanish National Health System. Carreras et al. [18], in a tertiary hospital in Barcelona, estimated that in 2019, potential drug cost avoidance represented 40% of pharmaceutical expenditures.

Another mechanism that may influence the economic implications of clinical trials is the research exemption to patent rights (often referred to as the “Bolar exception”), which allows the use of patented medicines within clinical trials without infringing intellectual property rights.

In conclusion, although there are large variations across type of tumor, overall, clinical trials performed at the medical oncology department of a tertiary university hospital are associated with high cost avoidance. Our results are consistent with those of other studies, suggesting that, in addition to early access to innovative therapies, the participation of hospitals in a national health system in oncology clinical trials could contribute to reducing hospital expenditures for cancer management.

## Supplementary Information

Below is the link to the electronic supplementary material.

Supplementary Material 1

## Data Availability

Data is available from the corresponding author upon reasonable request.

## Abbreviations

IQR: Interquartile range
RCT: Randomized controlled trial
