# Supplementary material for: Financial impact of participating in oncology clinical trials in a tertiary hospital in Spain
Source: BMC Health Serv Res. 2026 Jul 18;26:1413. doi: 10.1186/s12913-026-15148-z (PMC13673791; doi:10.1186/s12913-026-15148-z)
Supplement: Supplementary file 1 [file 12913_2026_15148_MOESM1_ESM.docx]

**Financial impact of participating in oncology clinical trials in a tertiary hospital in Spain**

**Sergio Mosquera-Ferrer^1^, Emilio Vargas-Castrillón^2^, Macarena Torrego-Ellacuría^3^**

^1^Clinical Pharmacology; Hospital Clínico San Carlos, Madrid, Spain

^2^Clinical Pharmacology; Pharmacology and Toxicology; Hospital Clínico San Carlos; Universidad Complutense de Madrid; Instituto de Investigación Sanitaria San Carlos (IdISSC), Madrid, Spain

^3^Innovation Unit, Hospital Clinico San Carlos, IdISSC, Madrid, Spain.

**Corresponding author**

Sergio Mosquera-Ferrer, PhD

Clinical Pharmacology

Hospital Clínico San Carlos

Calle del Prof Martín Lagos, S/N

28040 Madrid

Phone: +34913303000

e-mail: sergiomosqueraferrer@gmail.com

**Additional file**

**Supplementary Table 1.** Resource use: diagnostic tests

| **Complementary tests*** | **In-house** | **External** | **Total** |
| --- | --- | --- | --- |
| Laboratory test | 11,427 | - | 11,427 |
| Computed tomography | 565 | 2,112 | 2,677 |
| Gammagraphy | 461 | - | 461 |
| Radiography | 427 | - | 427 |
| Echography | 240 | - | 240 |
| PET-CT | 183 | - | 183 |
| Magnetic resonance | 180 | 167 | 347 |
| Catheterism | 115 | - | 115 |
| Biopsy | 42 | - | 42 |
| Densitometry | 28 | - | 28 |
| Other | 136 | - | 136 |

*All figures represent the number of tests.

Unit costs can be found in reference #19. However, note that diagnostic tests are presented in aggregated categories (e.g., laboratory tests and radiography). However, within each category, individual tests have different unit costs (for example, a hand radiograph and a chest radiograph have different prices). Therefore, reporting a single unit cost or an average cost for these aggregated categories would not be representative and could be misleading.

PET-CT positron emission tomography‒computed tomography

**Supplementary Table 2.** Resource use: medical visits

| **Visits*** | Number of visits | Unit cost (€) |
| --- | --- | --- |
| Chemotherapy day unit visits | 13,499 | 150,00 |
| Nursing visits | 5,195 | 21,00 |
| Follow-up medical oncologist visit | 1,284 | 79,00 |
| Telephone medical oncologist visit | 644 | 56,00 |
| Follow-up genetic counseling | 335 | 212,00 |
| First-visit genetic counseling | 15 | 212,00 |
| First medical oncologist visit | 9 | 132,00 |
| Hospital ward | 4 | 79,00 |

*All figures represent the number of visits.

**Supplementary Table 3.** Drug cost avoidance by tumor site

| **Type of tumor** | **Overall** | **Per patient** |
| --- | --- | --- |
| Non-small cell lung cancer | 5,698,206.43 | 53,254.27 |
| Ovarian cancer | 5,283,720.03 | 92,696.84 |
| Breast cancer | 4,379,542.17 | 27,895.17 |
| Renal cancer | 3,289,467.16 | 99,680.82 |
| Prostate cancer | 1,905,517.83 | 32,296.91 |
| Bladder cancer | 1,632,627.94 | 15,698.35 |
| Solid tumors | 829,347.92 | 10,238.86 |
| Liver cancer | 755,942.91 | 94,492.86 |
| Other | 701,310.57 | 14,610.64 |
| Melanoma | 505,875.00 | 45,988.64 |
| Colorectal cancer | 451,658.66 | 18,066.35 |
| Uterine cancer | 350,307.67 | 29,192.31 |
| Small cell lung cancer | 89,149.12 | 5,943.27 |
| Gastric cancer | 22,649.51 | 3,774.92 |
| Gallbladder cancer | 3,824.66 | 956.17 |

**Supplementary Figures**

**Supplementary Figure 1.** Clinical trials and patient disposition

**Supplementary Figure 2.** Distribution of trials by sponsors

The figure shows only sponsors who have conducted more than one trial.

**Supplementary Figure 3.** Cost of dispensed medications by tumor type
